# Enhancing Parents' Experiences of Paediatric Genomic Testing in Usual Outpatient Care: Insights From a Multi‐Perspective Qualitative Study

**DOI:** 10.1111/jpc.70538

**Published:** 2026-08-18

**Authors:** Erin Crellin, Melissa Martyn, Selima Ymer, Maya Pinn, Emma Weisz, Clara Gaff, Belinda Dawson‐McClaren

**Affiliations:** ^1^ Genomics in Society, Murdoch Children's Research Institute Melbourne Victoria Australia; ^2^ Department of Paediatrics The University of Melbourne Melbourne Victoria Australia; ^3^ Melbourne Genomics Health Alliance, Walter and Eliza Hall Institute Melbourne Victoria Australia; ^4^ Parent Project Advisor Melbourne Victoria Australia; ^5^ Monash Children's Hospital Melbourne Victoria Australia

**Keywords:** exome sequencing, health services research, paediatrics, parents, qualitative research, rare diseases

## Abstract

**Aim:**

The aim of this research was to explore what makes (or would make) for good genomic care delivered wholly or in part by paediatricians.

**Methods:**

An interpretive description, qualitative study with parents of children offered genomic testing as an outpatient for a condition other than cancer; general and subspecialist paediatricians; nurses; and genetic counsellors. Data were primarily collected retrospectively and analysed using inductive content analysis. Interpretation was enriched by the involvement of parent and paediatrician project advisors.

**Results:**

Twenty‐five parents and 20 health professionals participated. Most parents had received a genetic diagnosis for a child presenting with neurodevelopmental delay and/or epilepsy. Key aspects of good genomic care identified included: adopting a slow, multi‐appointment approach to discussing genomic testing in certain instances; letting families know what to expect; signposting to information and psychosocial support services when disclosing results; communicating next steps; and adopting a team approach to aid sense‐making. Paediatricians were not expected to do everything, with ongoing roles for genetic experts described. At different stages of the testing process, paediatricians existing and ongoing relationships with families were identified as an asset in helping promote good experiences.

**Conclusion:**

Genomic testing in usual paediatric outpatient care can be delivered well, with this research yielding practical guidance for paediatricians. Ongoing evaluation as models of care evolve will support the delivery of high‐quality genomic care that best meets families' needs.

## Brief Points

- What is already known on this topic
  ○ The availability of federal‐ and state‐funded genomic testing in paediatric outpatient settings is increasing.
  ○ There are many known barriers to integrating genomic testing into usual care; addressing these through tailored interventions is the focus of a burgeoning area of research
  ○ Understanding how care experiences can be enhanced is also essential, with practical guidance for paediatricians needed
- What this paper adds
  ○ Our findings suggest paediatricians' existing and ongoing relationships with families are an asset for promoting positive care experiences of genomic testing and may result in some well‐described needs being better met.
  ○ Key aspects of ‘good’ genomic care identified through engagement with parents, paediatricians and others included: adopting a slow approach in certain instances to support informed decision‐making; signposting to informational resources and support services when disclosing results; and multidisciplinary involvement to help families make sense of results.

## 1 Introduction

Genomic testing (defined herein as multigene panels, exome or genome sequencing) provides more accurate diagnoses for children with rare genetic conditions, and can inform management [1]. Moreover, federal‐ and state‐funded tests now exist that can be ordered by paediatricians without the need to refer families for a clinical genetics appointment first (see the Supporting Information for details about the Australian context) [2]. This aims to improve timely patient access to genomic testing and free up clinical genetics services for complex cases [3].

There are well‐described barriers to integrating genomic testing into usual outpatient care, defined herein as care typically provided by paediatricians (and other nongenetic health professionals) in outpatient settings. Addressing these barriers [4, 5, 6, 7, 8, 9, 10, 11, 12, 13, 14, 15] (summarised in Table 1) through tailored interventions is the focus of a growing area of research. For example, a program of work in Victoria recently investigated why requests for genomic testing for suspected childhood syndromes by general paediatricians were lower than anticipated, and how identified challenges could be best addressed [9]. Co‐designed interventions and awareness raising activities supported test use [16].

**TABLE 1:** Paediatrician‐reported barriers to using and navigating genomic testing.

Ambiguous patient eligibility criteria [5, 9]
Low confidence, knowledge (e.g., selecting the right patients, tests [4, 6, 7, 8, 14]; conducting consent conversations [9, 11]; interpreting and disclosing results [4, 6, 8, 10, 12], particularly variants of uncertain significance [7, 13])
Concerns about providing an inferior patient service [4, 9]
Perceived conflict with professional identity [9]
Burdensome processes and paperwork [4, 9, 10, 11]
Difficulties accessing genetics experts for advice [9, 10, 11]
Lack of time [4, 5, 6, 9, 10, 11, 15]
Lack of Medicare items for time spent on genomic testing‐related activities [9, 10]

Ensuring genomic test delivery promotes good care experiences is an important implementation consideration [17]. Paediatricians have raised concerns about providing a poor patient service [4, 9], which guidance on how care experiences can be enhanced may help address. Relatively little is known about how families experience genomic testing in usual paediatric care [17]. The limited research that has been conducted with parents has mostly centred on testing offered in the context of genomics research programs [18], or examined experiences at a single health service [19]. To guide service delivery, we explored parents' perspectives on what makes (or would make) for good care experiences when genomic testing is delivered as part of usual outpatient care. Health professionals' perspectives were secondarily sought to gain insight into shared understanding of good care and how parent needs may be met.

## 2 Methods

### 2.1 Methodology

An interpretive description approach was used in this qualitative study, an approach well‐suited to research aiming to inform clinical practice [20].

The research was also guided by pragmatic and participatory principles. The study design was responsive to the evolving genomics implementation landscape. Funding enabled parent advisors to join the research team mid‐project.

The Standards for Reporting Qualitative Research [21] guided reporting of the study design and findings.

### 2.2 Recruitment

Recruitment was national and aimed to capture a range of perspectives to provide a rich understanding.

#### 2.2.1 Parents

Study criteria included:

- *Genomic testing* offered for a child within the last 5 years in an *outpatient setting* for a *condition other than cancer*.
- By a *paediatrician*, that is a general paediatrician or non‐geneticist paediatric subspecialist (priority group 1) OR through a clinical genetics service (priority group 2)

Cancer was excluded due to the treatment‐focussed and complex nature of genomic testing in this setting (genetic changes in both tumour and germline cells are often examined).

The perspectives of some parents who had genomic testing via a clinical genetics service were purposively sought as a pragmatic means of exploring what a good service might look like when the adoption of a new practice remained limited [22]. These parents were asked to draw upon multiple experiences, including their experiences of interacting with a paediatrician.

The extensive strategies pursued to recruit parents are outlined in the Supporting Information.

#### 2.2.2 Health Professionals

Professional networks were used to recruit: general and subspecialist paediatricians with some experience ordering genomic testing (or an interest in doing so); nurses and genetic counsellors interacting with families offered genomic testing by paediatricians.

### 2.3 Data Generation and Analysis

Data were primarily collected retrospectively via focus groups and interviews conducted by video conferencing or phone by author EC. Both parents were present for the first half of one interview; however, the father remained mostly in the background. The other interviews involved one parent. Repeat interviews were conducted with three parents: one to ask some follow‐up questions; another to receive an update about their child's test request; and a third to hear their experience receiving their child's results. Focus groups/interviews were recorded, transcribed verbatim, de‐identified then analysed via inductive content analysis [23]. The bulk of the parent data were coded first. Further analysis occurred parallel to analysing the health professional data, with comparisons made within/between groups. See the Supporting Information for details.

## 3 Results

### 3.1 Participant Characteristics

#### 3.1.1 Parents

Twenty‐five parents (21 mothers, 4 fathers) of 25 patients participated between August 2023 and March 2025. Three parents via a one‐hour focus group; 21 via interviews averaging 37 min (range 12–72). Parent and patient characteristics are outlined in Table 2. Of note, neurodevelopmental delay ± epilepsy was the most common indication for genomic testing. Most parents received a diagnostic result for their young child.

**TABLE 2:** Parent and patient characteristics.

		*n*
*Parents interviewed* ^a^ (*N* = 25)		
Gender	Female	21
	Male	4
State	New South Wales	3
	Queensland	1
	South Australia	2
	Tasmania	1
	Victoria	16
	Western Australia	2
Region	Metropolitan	15
	Regional	10
*Patients* ^b^ (*N* = 25)		
Model of genomic care child/ren received	General paediatrician involved (extensive genomic test discussions ± test ordering)	6
	Paediatric subspecialist involved (extensive genomic test discussions ± test ordering)	14
	Referral to a clinical genetics service for testing	5
Clinical indication for testing	Neurodevelopmental delay ± epilepsy	20
	Isolated renal condition	2
	Multiple congenital anomalies, other complex presentations	3
Age of child when genomic testing offered	Under one	8
	1–2 years^c^	5
	3–4 years	2
	5–7 years	6
	8–11 years	2
Genomic test outcome^d^	Diagnosis	18
	No diagnosis	3
	Uncertain	1

^a^ Both parents were present for part of one interview. The father primarily remained in the background, chiming into the conversation occasionally.

^b^ Three parents interviewed had two children offered genomic testing for unrelated conditions. Both children included in this study for only of these parents. In the case of the other two, one child had testing as an inpatient and the other had testing when the family was living overseas.

^c^ One of these parents decided to go ahead with testing when their child was three.

^d^ Testing for three children was either still being actively deliberated by parents at time of interview, awaiting approval, or delayed due to state‐based authorisation issues.

#### 3.1.2 Health Professionals

Twenty health professionals were interviewed between September 2024 and February 2025. Interviews averaged 40 min (range 25–70). Six and four participants were general and subspecialist paediatricians, respectively; two were nurses involved in genomic testing discussions with paediatricians and families and follow‐up care; eight were genetic counsellors who worked with paediatricians (see Table 3). One general paediatrician, one nurse and the four subspecialists interviewed had extensive genomics experience (see Table S4 for further information).

**TABLE 3:** Health professional characteristics (*N* = 20).

		*n*
Profession	General paediatrician	6
	Paediatric subspecialist (not a clinical geneticist)	4
	Nurse	2
	Genetic counsellor	8
Location	New South Wales	3
	Queensland	2
	Victoria	14
	Western Australia	1
Practice setting	Public	18
	Private	1
	Public and private	1
Level of genomics experience^a^	None (interested but still referring patients to genetics)	1
	Some (involved with ordering a few genomic tests; beginning to support families navigating testing)	5
	Extensive (ordered many tests; genomics champion within specialty; leading support for families)	6

^a^ Genetic counsellors omitted here.

### 3.2 Categories

Illustrative quotes are presented in Table 4.

**TABLE 4:** Categories and illustrative quotes.

Category		Quote number	Quote
How informed decision‐making can be supported	By discussing testing with child's usual paediatrician	1	“It [genomic testing] was raised in a really sensitive and delicate way.… we had known our nephrologist for [X] years. So it wasn't like someone new kind of suggesting something unusual… we felt very trusting and supported at that time” (Parent 10, diagnostic result)
		2	“Already in [child's] appointment they were discussing, being a [general] paediatrician, discussing many different aspects of things that [child] was having issues with, so I guess it [genomics] was seen as a part of the picture of [child's] overall health. Well, that's how I saw it” (Parent 19, negative result)
	By communicating information simply & transparently; letting parents know what to expect	3	“We did have a really good experience.… making sure that they're [paediatricians are] explaining what the test is so that obviously families know exactly what's being looked at and… kind of like the process that they're probably looking at” (Parent 24, diagnostic result)
		4	“You're pretty much just left in the dark…if you had some idea of what's down the line sort of thing [in terms of genomic investigations] then yeah, it's better than walking in blind” (Parent 16, negative result)
	Genetic counsellors' concerns about quality of consent process	5	“I just can't see any paediatrician or neurologist having the time to give good patient service and experience.… I spend an hour with my families… part of that hour is getting medical information and a family history… but then a good half an hour is probably spent on genetic testing.… What can we do? … What would you expect if they [results] come back normal?” (GC08, private genetics clinic)
		6	“Often testing is presented as … we're going to do this test… to help us find XYZ. Sign here … the doctors are coming from a, well as a healthcare professional, I need to know… to help treat you, which is valid… it just doesn't allow for that conversation around, well, is now the right time for the family?” (GC04, embedded in a paediatric clinic)
	By adopting a slower approach in some circumstances	7	“It is quite confronting… I've always known [two‐year‐old child] didn't fit the normal box… to be told by a paediatrician that they are in the belief that might be it too… [there's] so much to take on and it may be that they spend one appointment talking to you about, OK, we're gonna do this [genomic testing] at your next appt, here is some information, take it away, read it” (Parent 22, testing delayed due to authorisation issues)
		8	“Our paed held our hands through that whole journey. We had so many conversations of like, ‘if we do whole exome, this is going to – this is what will happen. You get a diagnosis. You won't get a diagnosis, or you'll get something and we don't know what it is.’” (Parent 03, focus group, diagnostic result)
		9	“If it's saying it's informed consent, they [parents] actually need a chance to process, to discuss, to seek other opinions if they want to. But when we spring it on them and try and just turn it all through in one appointment, it often doesn't give them that chance.” (Paed 06, general paediatrician)
		10	“For nephrology, it's such a slow burn and chronicity thing that we've seeing a lot of our families until they are 18. So I just sort of like in my notes say look I've raised you know testing here; we discussed this. I've offered to re‐discuss it, you know, every couple of years” (Paed 08, paediatric subspecialist)
		11	“They [paediatricians] can have the conversation and say this is a potential, but you don't need it this year necessarily. Potentially you don't even need it next year, but this is something that is an option for you… you can really slow things up… they're in a prime position… to do that and they see them regularly” (GC05, working with general paediatricians)
	Using different approaches in different contexts	12	“We needed to know why the seizures were occurring … we needed to get on the right drug… I had no idea what was going on [when genomic testing was raised] and it was sort of going over my head. So at that moment when they were doing the swabs like… Could they have genetics come up [to the clinic] or… there's a real opportunity for like [condition] nurses or someone to come up… support the family” (Parent 06, diagnostic result)
		13	“My clinics are very booked out for months. I don't have the luxury to say have a think about this and I'll see you again in clinic. Sometimes if they want to think about it I can say they can, you know, contact us back again or something, but it tends to be just on the 1 day one appointment” (Paed 05, paediatric subspecialist)
What delivering results well looks like	Knowing what to expect and not disclosing results over the phone	14	“We weren't expecting to get them that day… it was a very big shock.… [With forewarning] … you could have emotionally or mentally prepared a little bit. Especially like we've got other kids as well. The meeting has gone so much longer… like oh my gosh, you gotta make sure this person that's looking after my kids knows that I'm gonna be… late and really upset when I pick them up” (Parent 14, diagnostic result)
		15	“If you… know the genetic test results back… OK, this kid has this… I've read through what this is, maybe then I should set up an appointment with a genetic counsellor… that'll give them time… to research it if they need to… so they can explain it better rather than just a parent getting [it] over the phone and diving into a deep, dark hole of depression trying to find… what everything means… it took me a solid 6 months to pull myself out of that hole” (Parent 05, diagnostic result)
	Scheduling a timely appointment (but challenging to enable)	16	“If you're telling the parents that there's a result, I think you need to be prepared to be able to actually tell them what the result is at that point in time or if not like the next day at the very most, the next week.” (Parent 23, diagnostic result)
		17	“If I want to see them, for instance, in a month's time to give them a result, that has to be an additional appointment in a clinic that's already overbooked… That's not ideal…[I]t's an unknown wait [for results]. If you could say, ‘look, the result will be back in 2 months. I will see you in 2 months and a day’, that would be helpful (Paed 03, general paediatrician)”
	Receiving results from child's usual paediatrician	18	“Being a person that you know and you feel comfortable with and can talk to… it is helpful to have them be the person giving it to you rather than this complete stranger… it also meant because [child's] quite comfortable with his paediatrician, it meant that it wasn't a stressful situation for [child], which means it wasn't then stressful for me.” (Parent 24, diagnostic result)
		19	“The things that made it a good, a positive experience with [child] was that we had a really stable medical team… well‐known to us, nephrologist and paediatrician” (Parent 07, negative result)
		20	“She [neurologist] should have [disclosed the result]. Because at the end of the day the geneticist did not add anything…with the neurologist there would have been … a plan, I guess, because when we did then have our next appointment with the neurologist [that's] sort of what happened… Leaving the geneticist, I was just sort of in shock…I'm just like, what do I even do?” (Parent 04, diagnostic result delivered by geneticist)
		21	“He [paediatrician] knows [child] and he knows the difficulties.… and that's what I meant by I guess like qualifying it [the genetic diagnosis] a bit more, like actually applying it to the person you can see in front of you” (Parent 17, diagnostic result delivered by geneticist)
	Speaking with an expert	22	“[doctor was] perfectly qualified to deliver this kind of thing … [They are a] genetics researcher [themselves]” (Parent 11, diagnostic result delivered by specialist with additional genetics expertise)
	Paediatrician confidence returning results varies according to type of result	23	“I've had one, two [patients] actually, when there's been a result, one of which… I was happy to do the initial, you know, we did these tests.… This is the finding… Genetics had with the result… listed a couple of reference papers, etcetera around it. So that gave me some background information to give them about the condition” (Paed 06, general paediatrician)
	Signposting parents to information resources, support services	24	“I expect [the doctor] to see the result, research themselves what that genetic disorder is…as much as we love our neuro that's not what she did.…had they done the research they could even say, hey, there's a Facebook page… feel free to join up that [group] and you know and talk to people…it is a lot for me … to do [the] researching” (Parent 21, diagnostic result)
		25	“They [paediatricians] should have… here's the list of psychologists here's the list of social workers… there's so much – like association for children with disability.… carer gateway, lots of places you can go for support” (Parent 03, focus group, diagnostic result)
		26	“I don't think you can expect the neurologist or the paediatrician to do it all… but in the same way that they would refer to cardiology to do the echo knowing… if there are psychosocial needs that there are support groups or telephone counselling services or… mental healthcare plans with the GP” (GC02, embedded in a paediatric clinic)
	Being told about next steps and facilitating parent understanding for all result types	27	“We were told that there was very likely nothing significant genetically going on… there may not be, but to have that gene variation pop up and then have no one to explain it. What? What in the world is going on? There needs to be some system there or at least a plan for like… Okay a quick Google says that it could be relevant. So you need to go see a genetic counsellor” (Parent 15, uncertain result)
		28	“Quite often you hand the family a result with a VUS [variant of uncertain significance] and say none of this is relevant. The results are negative, at this stage… you do have to be kind of simplify it a little bit. That doesn't mean that in the future we won't find something” (Nurse 01)
Follow‐up helps parents make sense of results	Paediatricians & allied health professionals aid sense making; putting results into context of whole child	29	“The thing that he [paediatrician] did well is being able to be a soundboard… I'd often end up waiting for when we've booked an appointment [to ask questions]. But then going into the appointment… I was able to ask him the questions… if he didn't have the answer … being able to guide us to where to find those answers …in the time of getting [child's] diagnosis… having quite regular appointments did really help” (Parent 24, diagnostic result)
		30	“It was really good to talk with the paediatrician about what the [genetic condition] might mean for other areas to do with everyday stuff like school and sport and learning and friendships and stuff… it was really good to have that avenue to talk more about what it meant for [child], in the context of the whole child” (Parent 10, diagnostic result)
		31	“Like every appointment we see them say… Did you think of any questions and maybe go through it [the genetic diagnosis] again… do you have any questions, just starting off each time?” (Paed 02, general paediatrician)
		32	“It was the first time our paediatrician had seen this variant…it's actually been the therapists that have been able to kind of give us more of those answers. Like you know… with walking… Do you think she'll ever walk? And then they go, yes, I think she will walk [laughs]…in situations like that… your allied health team is gonna be your everyday backbone… because you're seeing these therapists every week, every fortnight” (Parent 23, diagnostic result)
	Roles for nurses	33	“If I could just throw something at someone and then they say yes, that's a really great question. Go to this resource … do this… and then I'll check in with you next week …if they can offer a nurse …for that particular presentation or group of presentations, then they're worth their weight in gold” (Parent 06, diagnostic result)
		34	“They have a dedicated nurse on that team now. She started a few months after [child] was diagnosed …now they do actually call and check in with parents pretty regularly after diagnosis… doctors are good, but they're not usually available to just have a long chat on the phone with the patient for checking in on them” (Parent 12, diagnostic result)
		35	“The role of the nurse is to… really understand the psychosocial dynamics of that family and repackage that information in a time and a place that ensures that it lands for that family…the sort of bridge between the genetic finding … to what it means for that family in their setting. Day to day does it mean NDIS… does it mean other clinical appointments” (Nurse 02)
	Genetic counselling valuable for detailed genetics information and family planning	36	“It was really great conversation with the genetic counsellor. He was able to answer lots of questions that we had about how do we find out if one of us has… passed [variant] on … if … any of our other children [were] at risk of carrying it …or having it. So that was all answered pretty quickly and then we were referred to have some genetic testing ourselves” (Parent 10, diagnostic result)
		37	“[genetic counsellor] will do some follow up for us… through a formal genetics appointment… family follow up and cascade testing and all that sort of thing. So that's a lot off my plate” (Paed 10, paediatric subspecialist)

### 3.3 How Parental Informed Decision‐Making Can Be Supported

#### 3.3.1 By a Familiar Paediatrician Discussing Genomic Testing and Letting Parents Know What to Expect

Parents often reported feeling well‐supported discussing genomic testing with a familiar general/subspecialist paediatrician (Table 4, Q1), despite some genetic counsellors' concerns about the quality of the consent process (Table 4, Q5–Q6). One parent described how their child's general paediatrician wove genomics into discussions about various aspects of their child's health (Table 4, Q2). Communicating information simply and transparently was valued, especially regarding what to expect throughout the testing process (Table 4, Q3). Parents who described more negative experiences often identified the latter as a key area for improvement (Table 4, Q4).

#### 3.3.2 By Adopting a Slow Approach in Some Circumstances

Several parents indicated a need for paediatricians to spread out information delivery and discuss genomic testing at more than one time point to aid decision‐making and information processing. These parents often had young children and were still adapting to having a child with a disability at the time in which testing was offered (Table 4, Q7). One parent who experienced a slow approach praised the way their young child's general paediatrician engaged in an ongoing discussion (Table 4, Q8).

Some paediatricians reported finding a slow approach helpful when genomic testing was non‐urgent (Table 4, Q9–Q10). The potential for discussions to be ongoing across appointments was considered a strength of embedding genomics into usual care (including by some genetic counsellors working with general paediatricians; Table 4, Q11), empowering families to proceed when the timing felt right.

It was evident a slow approach would not be appropriate or feasible in all situations; however (Table 4, Q12–Q13).

### 3.4 What Delivering Results Well Looks Like

#### 3.4.1 Balancing Forewarning With Minimising Worry

Many parents stressed the importance of knowing when and how results will be returned. Negative experiences of being caught off‐guard were often described, with results returned unexpectedly by phone or at what was anticipated to be a routine appointment (Table 4, Q14–Q15). On the flipside, a few parents were unfazed receiving results without warning and thought—if parents were to be notified results are back—the gap between doing so and fitting parents in for an appointment should be brief to minimise worrying time (Table 4, Q16). Interviews with paediatricians shed light on some of the challenges associated with scheduling results appointments when test turnaround times are uncertain and clinic appointments scarce (Table 4, Q17).

#### 3.4.2 Receiving Results From a Familiar, Trusted Paediatrician

Parents often reported that receiving their child's results from a familiar, trusted paediatrician enhanced their experiences (Table 4, Q18–Q19). Further, some parents who received their child's results from a clinical geneticist indicated they would have preferred to have received them from their child's usual doctor. These parents felt that their child's paediatrician would have helped them better understand what the results practically meant and leave with a plan for next steps (Table 4, Q20–Q21). Conversely, a few parents valued speaking with a geneticist (Table 4, Q22), particularly when results were complex.

Paediatricians' reported confidence returning diagnostic results varied according to the nature of the result (e.g., single vs. multi‐system impacts). One general paediatrician described how information resources from the clinical genetics service helped them take on some results disclosure (Table 4, Q23).

#### 3.4.3 Being Prepared to Signpost Parents to Information and Support Services

Parents felt it was essential for paediatricians to briefly research what condition‐specific information resources and supports are available ahead of disclosing results (Table 4, Q24). Written resources to take away, when provided, were highly valued. Many parents reported not being directed to support services (e.g., mental health, carer supports; genetic support groups), with adverse impacts when this was missed. Parents, genetic counsellors and nurses did not expect paediatricians to provide all the support; rather, recognise the need for psychosocial support and signpost families to groups and services (Table 4, Q25–Q26).

#### 3.4.4 Providing Clarity on Next Steps

All parents stressed the importance of helping parents understand results and communicating a plan for next steps, irrespective of whether their child received a diagnosis or not (Table 4, Q27). These views were echoed by one nurse interviewed and many genetic counsellors (Table 4, Q28).

### 3.5 Follow‐Up Helps Parents Make Sense of Results

Parents emphasised the importance of follow‐up. Making sense of what a result means for one's child and family was an ongoing process, and parents valued and indicated a need to have the option to engage with different health professionals to help them do so.

#### 3.5.1 Paediatricians, Allied Health Professionals and Nurses Aid Sense‐Making

Several parents cited examples of how their child's paediatrician aided sense‐making. For instance, one parent described how their child's general paediatrician served as a ‘sound[ing] board’ in the months following results disclosure, with the ability to raise questions that arose with time at subsequent appointments and engage in an ongoing dialogue much valued (Table 4, Q29). Another, who initially received their child's result from a paediatric subspecialist, voiced it was their child's general paediatrician who helped them make sense of what the result meant for their child as a ‘whole’ (Table 4, Q30). Some general paediatricians interviewed also saw their ongoing relationships with families as a potential asset for Supporting Information processing (Table 4, Q31).

A few parents reported that involving their child's therapy team was invaluable for making sense of what a result practically meant for their child. Indeed, these parents indicated their child's therapists became the main outlet for their questions over time (Table 4, Q32).

Some parents also identified roles for nurses in aiding sense‐making, checking in on families post results disclosure, and being a point of contact for questions (Table 4, Q33–Q34). Nurses interviewed reported beginning to help families process genomic results and navigate next steps (Table 4, Q35).

#### 3.5.2 Genetic Counselling Valuable for Navigating Familial Impacts and Further Genetic Information

To make sense of what a result may mean for future pregnancies and the wider family, parents and paediatricians agreed genetic counsellor involvement was valuable (Table 4, Q36–Q37). Genetic counsellors were also considered well placed to answer parents' genetic‐specific questions; ensure parents have ‘full information’; and respond to parents' immediate psychosocial support needs. A few parents additionally described how establishing a link with a genetic counsellor was helpful for learning the latest information and research opportunities down the track.

Parents also indicated a need for longer‐term psychological support from counsellors or psychologists experienced in childhood disability/illness to help families process results. None of the parents who raised this reported receiving such support; however.

## 4 Discussion

This study offers practical insights for paediatricians on what they can do to enhance parents' experiences of genomic testing in outpatient settings. Paediatricians are known to find genomic testing daunting and express concerns about their ability to provide a good service for families [4, 9]. Our findings on what makes for good genomic care may thus be reassuring in that they largely mirror what is known to be important for delivering a disability or other paediatric diagnosis well [24, 25]. Doing what paediatricians do well in other areas of practice will likely stand them in good stead. Their existing and ongoing relationships with families were also an evident asset, echoing findings from a recent paediatric immunology study [19]. Indeed, our research points to opportunities for parents' experiences to be enhanced by integrating genomic testing into usual care, with follow‐up a good example.

Follow‐up is a well‐described unmet need in the genetics literature [17]. Our data suggest that there are aspects of follow‐up paediatricians are well‐placed to lead. Paediatricians' ongoing relationships with families allow for emerging questions to be discussed over time, and meaning to be co‐constructed in the process. Paediatricians' knowledge of the child and family was also seen as a strength for helping parents put results into context (see Table 4, Q21, Q30).

Opportunities for enhanced care were also evident at the pre‐testing stage. Some parents valued the supportive environment afforded by genomic testing being raised by familiar, trusted doctor. Weaving genomic testing into usual care discussions was also shown to have the potential to normalise genetics as ‘part of the picture’ (Table 4, Q2), rather than genetics being seen as something intimidating or scary [26]. There was also evident potential for testing discussions to unfold over time. The value of multiple conversations in supporting informed decision‐making for some families has previously been recognised in acute care contexts, where rapid test results are required [27]. Our research extends the discussion of where this strategy may be helpful to outpatient settings, where there is less urgency. As genomic testing becomes a standard of care and is offered earlier in the diagnostic pathway, families will increasingly still be in the throes of adapting to having a child with additional needs (Table 4, Q7). Building in time for families to process information and decide if the timing feels right will thus likely become all the more important.

Paediatricians may also find it reassuring to hear that they were not expected to do everything. Indeed, there was evident value in a multidisciplinary approach following results disclosure. Genetic experts remain best placed for managing familial and reproductive implications and complex results, as recognised in a recently developed framework describing models of non‐geneticist involvement in genomic testing [28]. Our findings also point to roles for other health professionals in augmenting the care paediatricians provide. Young children presenting with neurodevelopmental delay may have a therapist team in place by the time genomic testing is initiated. Involving these therapists in the result sense‐making process (e.g., by deferring questions related to tasks of daily living, acquisition of skills) will likely be valuable. Roles for nurses in supporting the delivery of genomic care have long been recognised [29]. Although discussion among participants primarily focused on hospital‐based nurses, thought should be given as to how nurses in other settings can incorporate genomics into their role. Communication between health providers will be key to the success of a team‐based approach. Understanding how communication can be enhanced as health professionals assume different roles would be valuable.

As it becomes more common for genomic testing to be initiated outside genetics services, it will be vital to continually evaluate what is/is not working well (from a range of perspectives) to drive service improvements [28]. Tailored measures will be needed to do so. Our initial data on what makes for good genomic care in outpatient settings could be leveraged to help develop quality indicators or a parent‐reported experience measure. Involving clinicians alongside families in the development of service evaluation tools will be essential to ensuring they are relevant and useful.

The findings of this exploratory study should be considered alongside its limitations. While the parent dataset was considered as a whole, the different data collection methods used (focus groups vs. interviews; one couple interview) may have yielded different types of responses. Most parents had experience of genomic testing for a child presenting with neurodevelopmental delay and/or epilepsy. While this limits the generalisability of our findings to other clinical contexts, neurodevelopmental presentations are ‘among the most common indications’ for paediatric genomic testing [1, p. 2029]. Parent accounts may have been impacted by recall bias, with the testing year range deliberately wide to facilitate recruitment. To further inform best practice, it would be helpful to prospectively interview families at different stages of the testing journey (e.g., while they are awaiting their child's results) in the future. Despite the considerable time and effort invested in parent recruitment, few parents had received an uninformative or uncertain result from genomic testing. Additional aspects may be gained from understanding if and how families' experiences differ when a diagnosis is not forthcoming. Understanding paediatricians' experiences discussing uninformative or uncertain genomic results with families would be equally valuable. In light of well‐established barriers, assessing competency and confidence undertaking different tasks in future studies would shed light on the quality of genomic care provided. All that said, examining diverse perspectives was a methodological strength, yielding a rich understanding of parents' needs and insight into feasibility considerations.

## 5 Conclusion

Our guidance for paediatricians (summarised in Table 5) will support good delivery of genomic testing in outpatient care. Parent experiences can be enhanced by applying skills paediatricians already have to genomic care and multidisciplinary involvement after results disclosure. This work complements the development of interventions to lower the barriers to paediatrician use of genomics. Ongoing evaluation as care models evolve will ensure genomic care best meets families' needs.

**TABLE 5:** How paediatricians can enhance parents' experiences of genomic testing.

Supporting informed decision‐making	Use plain language and be open about what the testing process involves, including the often long wait for results.
	Apply a slow (multi‐appointment) approach where needed/feasible. Parents of young children especially may be overwhelmed by other issues when testing is first raised. Provide information for parents to take away and digest and allow for genomic testing discussions to unfold and be revisited over time.
Returning results well	Co‐develop a plan with parents for returning results. Parents have found it distressing to receive results unexpectedly, particularly by phone and without a support person present.
	Undertake initial results disclosure where possible; parents value receiving results from someone they know and trust. Briefly research diagnostic results before the appointment and/or consult with a clinical genetics service.
	Provide condition‐specific written information for families to take away and process in their own time.
	Be aware of, and signpost families to, psychosocial supports. For example: Peer support for families with children with diagnosed or undiagnosed rare genetic conditions available through Syndromes Without a Name, www.swanaus.org.au Community supports and genetic support groups compiled by Genetic Alliance Australia, www.geneticalliance.org.au; The Genetic Support Network of Victoria, www.gsnv.org.au Mental health and well‐being supports collated on the RARE Portal, www.rareportal.org.au
	Communicate a plan for next steps for all results. Offer follow‐up appointment(s) as an opportunity to ask further questions.
Facilitating sense‐making	Check‐in with parents and create space for questions at subsequent appointments. Parents value discussing their child's result further with someone who knows their child well and can help put the result into context.
	Adopt a multidisciplinary approach to help parents make sense of results. For example, by: Involving a child's usual therapists—some parents report them to be a valuable outlet for their practical questions Referring families for genetic counselling

## Author Contributions

Conceptualisation: Erin Crellin, Melissa Martyn, Clara Gaff, Belinda Dawson‐McClaren. Funding acquisition: Erin Crellin, Clara Gaff. Methodology: Erin Crellin, Melissa Martyn, Clara Gaff, Belinda Dawson‐McClaren. Investigation: Erin Crellin, Melissa Martyn. Formal analysis: All. Supervision: Melissa Martyn, Clara Gaff, Belinda Dawson‐McClaren. Writing – original draft: Erin Crellin. Writing – review and editing: All.

## Funding

E.C. was funded by a National Health and Medical Research Council (NHMRC) (GNT2005257) Postgraduate Research Scholarship, a Murdoch Children's Research Institute top‐up scholarship, and a June Opie fellowship from The University of Auckland. Funding from the Melbourne Genomics Health Alliance facilitated parent data collection. Work undertaken at the Murdoch Children's Research Institute was supported by the Victorian Government's Operational Infrastructure Support Program. The contents are solely the responsibility of the individual authors and do not reflect the views of the NHMRC.

## Ethics Statement

The study was conducted under an approved human research ethics committee protocol, with approval granted by The University of Melbourne (HREC: #25541).

## Conflicts of Interest

The authors declare no conflicts of interest.

## Supporting information


**Table S1:** Examples of Medicare‐funded paediatric genomic tests that now exist in Australia.
**Table S2:** Parent recruitment strategies pursued.
**Table S3:** Topics canvassed with health professionals during interviews.
**Figure S1:** Overview of the analysis process.
**Table S4:** Genomics experience levels of paediatricians and nurses.

**Data S1:** Standards for Reporting Qualitative Research (SRQR)*.

## Data Availability

The data that support the findings of this study are available from the corresponding author upon reasonable request.
