# Supplementary material for: Enhancing Parents' Experiences of Paediatric Genomic Testing in Usual Outpatient Care: Insights From a Multi‐Perspective Qualitative Study
Source: J Paediatr Child Health. 2026 Aug 18;62(10):1959–70. doi: 10.1111/jpc.70538 (PMC13671241; doi:10.1111/jpc.70538)
Supplement: Supplementary file 2 [file JPC-62-1959-s001.docx]

# Supporting Information

## Healthcare and genomic testing landscape in Australia

Australia has a federated, mixed public/private healthcare system. Public health services are provided at a state and territory level through a combination of federal (Medicare) and state funding.

Australian paediatricians are secondary or tertiary healthcare providers. The paediatrician training program firstly involves three years of basic training.^1^ Trainees then undertake three years of advanced training in general or community paediatrics (qualifying as a general or developmental paediatrician) or a given subspecialty.^2^

A number of genome-wide sequencing tests (‘genomic tests’) are now funded federally through Medicare (see Table S1 for examples).^3–5^ In some tertiary hospitals paediatricians can also request certain genomic tests through the hospital’s pathology department. Requests are reviewed by an expert panel. The increased availability of federal- and state-funded genomic tests is the result of national and state genomics research programs that have demonstrated the clinical and cost effectiveness of genomic testing for different patient populations.^6^ Federally, the Medicare Services Advisory Committee reviews available evidence when making funding decisions about genomic testing and other health technologies.^7^

Table S1. Examples of Medicare-funded paediatric genomic tests that now exist in Australia

| **Clinical context** | **When Medicare item/s became available** |
| --- | --- |
| Childhood syndromes | May 2020 |
| Kidney disease | July 2022 |
| Neuromuscular diseases | November 2022 |
| Childhood hearing loss | November 2023 |
| Mitochondrial disease | November 2023 |

## Recruitment

### Parent recruitment strategies

Extensive strategies were used to recruit parents (see Table S2). By far, the most successful strategy was parent advisors posting information about the study on online support groups, with 16 of the 24 parent participants recruited via this avenue. Considerable effort was made to try and recruit parents who had received different types of results (i.e. diagnostic, uninformative). For example, by recruiting through specific and broad support groups; displaying flyers in hospital waiting rooms. Mail outs also deliberately targeted families who had received informative and uninformative results.

Table S2. Parent recruitment strategies pursued

| **Strategy** | **Examples** |
| --- | --- |
| Broadcast | - Advertising study at various meetings/events (e.g. clinical department meetings, Genetic Support Network of Victoria wellness series) - Advertising study in article about paediatric genomic testing written for Source Kids^8^ - Displaying study flyers in clinic waiting rooms, hospital noticeboards (VIC only) |
| Support groups (researcher-led) | - Posting study flyer on various genetic and disability online support groups (e.g. Rare Voices Australia, Too-peas-in-a-podcast hangout, Genetic Alliance Australia, developmental delay support groups), often at multiple time points - Study information also included in some support group newsletters (e.g. Syndromes Without a Name, Leukodystrophy Australia) |
| Support groups (parent advisor-led) | - Posting study flyer on various genetic and disability online support groups at multiple time points (e.g. Feeding Tube Australia, Epilepsy Australia, global developmental delay support group for parents, NAPA Centre, Pitt Hopkins syndrome parent support group) |
| Clinician-led | - General paediatricians, paediatric subspecialists, genetic counsellors in professional network sharing study flyers with families |
| Mail out | - Targeted mail out with three VIC-based hospital paediatric clinics delivering genomic testing to children and families |

### Sample size

Pragmatic considerations (e.g. time available) and the concept of information power^9^ informed decisions around when to stop parent and health professional recruitment. Information power is determined by a range of factors, including the richness and range of participant accounts^.9^

In line with the exploratory nature of our study design, we identified different groups of health professionals to recruit through the course of our interviews. We reflected on the information gained from each interview when considering whether further recruitment was worthwhile. Upon determining we had gathered sufficient insights to understand the care domains and topics raised in parent interviews, we pragmatically ceased recruitment.

## Data generation

### Parents

#### Online focus groups

To prompt discussion among parents on what a good service might look like when genomic testing is delivered by paediatricians, deliberative online focus groups^10^ were initially pursued. This approach involved:

- Opening with an icebreaker activity to build rapport
- Presenting a short video summarising what past research^11^ suggests makes for good service experiences of genetic testing (most parents in these studies had testing through clinical genetics services)
- Facilitating guided discussion by asking parents: what they thought of the care factors described in the video; what they would want or expect a paediatrician to do; benefits and challenges of greater paediatrician involvement in delivering genomic testing; what would be important for parents to feel supported.

Author EC moderated the discussion, with the support of MM (an experienced health service researcher with expertise in qualitative methods). EC is a PhD candidate with previous experience in the use of qualitative methods. EC also has lived experience of childhood-onset disability. Some parent participants were aware of this, with benefits to the ease and flow of the conversation noted. EC remained mindful of the possible influence of her background on parent interactions and data interpretation through regular journalling and supervision.

One week following the focus group, parents were invited to share any further reflections via email.

While this method was found to be helpful for exploring how experiences can be enhanced at an early stage of an innovation being embedded into usual care, focus groups proved very difficult to organise with time-poor parents.

#### Semi-structured interviews

Switching to semi-structured interviews coincided with increased recruitment of parents from priority group 1. As interviews involve 1-1 interactions, the responses may differ from focus groups where participants interact with each other as well as the researcher.

Interviews were conducted by EC using a flexible interview guide with prompts.^12^

Broad topics for priority group 1 included:

- Experiences of discussions about genomic testing/pre-test counselling
- Experiences waiting for genomic test results
- Experiences receiving genomic test results

At each of these stages, parents were asked about what they found helpful/unhelpful in terms of service delivery; what they expected or wanted paediatricians to do; and possible ways for families to be better supported in the future.

Similar topics to the focus group were covered for priority group 2, with the video omitted.

Three parents were interviewed on two occasions. Two of the three repeat interviews were conducted with parents who were still waiting for their child’s test results at the time of the first interview. For one of these parents, genomic testing for their child did not go ahead even though they gave their informed consent and went through the sample collection process. Their child’s general paediatrician’s request was reportedly denied due to an issue in who was deemed eligible to request an exome in their State.

All parents – regardless of whether they participated in a focus group or interview – received a $30 gift voucher as a token of appreciation of their time. Parents provided written consent, with all informed about how their data would be used and stored.

### Health professionals

Interviews with health professionals were conducted by EC using a flexible interview guide tailored to the profession interviewed. Broad topics canvassed are included in Table S3. Health professionals provided written consent to be interviewed, with all informed about how their data would be used and stored.

Table S3. Topics canvassed with health professionals during interviews

| Common topics: practice setting; key questions health services should ask families about their experiences of genomic testing | |
| --- | --- |
| Paediatrician-specific topics | - Experiences discussing and ordering genomic testing - Strengths and challenges in greater paediatrician involvement in delivering genomic testing, in terms of how families might experience care - Key things paediatricians can do to enhance families’ experiences of genomic testing; roles for other health professionals |
| Nurse-specific topics | - Nature of involvement with delivering genomic testing - Possible roles for nurses in supporting families at different stages of the testing process - Parent needs nurses may be well-placed to serve - Patient-centred genomic care (what should/can it look like?) |
| Genetic counsellor-specific topics | - Views on how families are experiencing genomic care provided by paediatricians - Key things paediatricians can do to enhance families’ experiences of genomic testing - Patient-centred genomic care (what should/can it look like in paediatric specialties?) |

## Data processing

All transcripts were transcribed verbatim by a research assistant. Author EC independently verified the accuracy of the transcripts against the focus group/interview audio recording. Transcripts were de-identified during this verification process.

## Data analysis

Focus group and interview data were analysed using a combination of inductive content analysis^13^ and methods championed in interpretive description.^14^ A summary is shown in Figure S1.

Figure S1: Overview of the analysis process.

Reflecting the different stages of data collection, the bulk of the parent data were analysed first, with further analysis then occurring parallel to analysing the health professional data.

EC listened to the audio recordings and read the de-identified transcripts multiple times to become deeply familiar with the data. Broad codes were then assigned to ‘chunks’ of text in each transcript in Microsoft Word by comparing and contrasting text within and between transcripts. Finer codes were subsequently generated and progressively refined. Memos were written during the coding process to encourage “analytical thinking”, ^14(p162)^ and emerging thoughts were regularly discussed with MM and BDM. Constant comparisons within and between parent and health professional groups helped group related codes into tentative categories, with these categories refined through discussion. Feedback from and discussion with parent advisors and a general paediatrician affiliated with the project was particularly valuable for testing out and refining ideas.

### Trustworthiness

Several techniques were used to enhance the trustworthiness and credibility of data analysis,^15^ including:

- Documenting decisions made and evolution of thinking via an extensive audit trail;
- Involving parent advisors and a general paediatrician affiliated with the project to aid data interpretation;
- Comparing and contrasting diverse stakeholder perspectives to provide a rich and more nuanced understanding of what constitutes good genomic care and feasibility considerations in delivering it.

## Participant characteristics

Table S4. Genomics experience levels of paediatricians and nurses

|  | **Participant** | **Genomics experience level** |
| --- | --- | --- |
| **Paediatricians** | Paed 01, general paediatrician | *Extensive*. Regularly ordering genomic tests since Medicare items became available. Genomics educator. |
|  | Paed 02, general paediatrician | *Some*. Ordered a few genomic tests in partnership with a clinical geneticist, returned some results. |
|  | Paed 03, general paediatrician | *Some*. Ordered a few genomic tests, returned some results. Self-reported confidence varied according to the nature of the results. |
|  | Paed 04, subspecialist paediatrician | *Extensive*. Regularly ordering state-funded genomic tests, returning results. Genomics education/training undertaken described during interview. |
|  | Paed 05, subspecialist paediatrician | *Extensive*. Regularly ordering state-funded genomic tests, returning results. Genomics education/training undertaken described during interview. Self-reported confidence varied according to the nature of the results. |
|  | Paed 06, general paediatrician | *Some*. Ordered a few genomic tests, returned some results. Self-reported confidence varied according to the complexity of the family’s needs and nature of the results. |
|  | Paed 07, general paediatrician | *Some*. Ordered a few genomic tests, yet to return results. Genetic rotation undertaken during paediatrician training. |
|  | Paed 08, subspecialist paediatrician | *Extensive*. Regularly ordering genomic tests since Medicare items became available. Genomics education/training undertaken described during interview. Self-reported confidence varied according to the nature of the results. |
|  | Paed 09, general paediatrician | *None*. Interested in learning how to independently request genomic testing for some patients. Experienced requesting genetic tests. |
|  | Paed 10, subspecialist paediatrician | *Extensive*. Genomics researcher; regularly ordering genomic tests since Medicare items became available. |
| **Nurses** | Nurse 01 | *Extensive*. Previously involved with genomics research programs. Involved in genomic testing discussions alongside subspecialist paediatricians and families; following up with families after results disclosure appointments. Up skilling undertaken described in interview. |
|  | Nurse 02 | *Some*. Involved in genomic testing discussions alongside subspecialist paediatricians and families; following up with families after results disclosure appointments. Interested in furthering genomic knowledge and counselling skills. |

## References

1.	The Royal Australasian College of Physicians. Paediatrics & Child Health. 2026. Accessed March 26, 2026. <https://www.racp.edu.au/trainees/basic-training/paediatrics-child-health>

2.	The Royal Australasian College of Physicians. Advanced Training. 2026. Accessed March 26, 2026. <https://www.racp.edu.au/trainees/advanced-training>

3.	Mallett AJ, Ingles J, Goranitis I, Stark Z. Implementation of reimbursement for genomic testing in Australia: early successes and the pathway ahead. *Intern Med J*. 2024;54(4):531-534. doi:10.1111/imj.16369

4.	Sachdev R, Field M, Baynam GS, et al. Paediatric genomic testing: Navigating Medicare rebatable genomic testing. *J Paediatr. Child Health*. 2021;57(4):477-483. doi:10.1111/jpc.15382

5.	Australian Government Department of Health and Aged Care. Genetic testing for neuromuscular disorders - new pathology MBS items 73422 to 73428. 2022. Accessed April 1, 2026. [http://www.mbsonline.gov.au/internet/mbsonline/publishing.nsf/Content/Factsheet- New-Path-items-73422-to-73428](http://www.mbsonline.gov.au/internet/mbsonline/publishing.nsf/Content/Factsheet-%20New-Path-items-73422-to-73428)

6.	Stark Z, Boughtwood T, Haas M, et al. Australian Genomics: Outcomes of a 5-year national program to accelerate the integration of genomics in healthcare. *Am. J. Hum. Genet*. 2023;110(3):419-426. doi:10.1016/j.ajhg.2023.01.018

7.	Norris S, Belcher A, Howard K, Ward RL. Evaluating genetic and genomic tests for heritable conditions in Australia: lessons learnt from health technology assessments. *J Community Genet*. 2022;13(5):503-522. doi:10.1007/s12687-021-00551-2

8.	Crellin E. Talking DNA: the difference genomics makes. *Source Kids*. 2024;(38):67. Available from: <https://sourcekids.com.au/source-kids-magazine-autumn-issue-2024/>

9.	Malterud K, Siersma V, Guassora AD. Information power: Sample content and size in qualitative studies. In: Camic P, ed. *Qualitative Research in Psychology: Expanding Perspectives in Methodology and Design*. 2nd edn. American Psychological Association; 2021:67-81. <https://doi.org/10.1037/0000252-004>

10. Rothwell E, Anderson R, Botkin JR. Deliberative discussion focus groups. *Qual Health Res*. 2016;26(6):734-740. doi:10.1177/1049732315591150

11. Crellin E, Martyn M, McClaren B, Gaff C. What matters to parents? A scoping review of parents’ service experiences and needs regarding genetic testing for rare diseases. *Eur J Hum Genet*. 2023;31:869-878. doi:10.1038/s41431-023-01376-y

12. Braun V, Clarke V. *Successful Qualitative Research: A Practical Guide for Beginners*. 1^st^ edn. London: SAGE Publications Ltd; 2013.

13. Vears D, Gillam L. Inductive content analysis: a guide for beginning researchers. *FoHPE*. 2022;23(1):111-127. doi:https://doi.org/10.11157/fohpe.v23i1.544

14. Thorne S. *Interpretive Description: Qualitative Research for Applied Practice*. 2nd ed. New York: Routledge; 2016. doi:10.4324/9781315545196

15. Liamputtong P. *Qualitative Research Methods*. 5th edn. Australia: Oxford University Press; 2020.
